# Core Hunter II: fast core subset selection based on multiple genetic diversity measures using Mixed Replica search

**DOI:** 10.1186/1471-2105-13-312

**Published:** 2012-11-23

**Authors:** Herman De Beukelaer, Petr Smýkal, Guy F Davenport, Veerle Fack

**Affiliations:** 1 Department of Applied Mathematics and Computer Science, Faculty of Sciences, Ghent University, Krijgslaan 281, S9, 9000 Gent, Belgium; 2 Department of Botany, Faculty of Sciences, Palacký University, Slechtitelu 11783 71 Olomouc, Czech Republic; 3, Bayer CropScience NV, Seeds, Technologiepark 38, 9052 Zwijnaarde, Belgium

**Keywords:** Core collections, Core subset selection, Genetic distance, Allelic diversity, Germplasm, Local heuristics, Multi-objective optimization

## Abstract

**Background:**

Sampling core subsets from genetic resources while maintaining as much as possible the genetic diversity of the original collection is an important but computationally complex task for gene bank managers. The Core Hunter computer program was developed as a tool to generate such subsets based on multiple genetic measures, including both distance measures and allelic diversity indices. At first we investigate the effect of minimum (instead of the default mean) distance measures on the performance of Core Hunter. Secondly, we try to gain more insight into the performance of the original Core Hunter search algorithm through comparison with several other heuristics working with several realistic datasets of varying size and allelic composition. Finally, we propose a new algorithm (Mixed Replica search) for Core Hunter II with the aim of improving the diversity of the constructed core sets and their corresponding generation times.

**Results:**

Our results show that the introduction of minimum distance measures leads to core sets in which all accessions are sufficiently distant from each other, which was not always obtained when optimizing mean distance alone. Comparison of the original Core Hunter algorithm, Replica Exchange Monte Carlo (REMC), with simpler heuristics shows that the simpler algorithms often give very good results but with lower runtimes than REMC. However, the performance of the simpler algorithms is slightly worse than REMC under lower sampling intensities and some heuristics clearly struggle with minimum distance measures. In comparison the new advanced Mixed Replica search algorithm (MixRep), which uses heterogeneous replicas, was able to sample core sets with equal or higher diversity scores than REMC and the simpler heuristics, often using less computation time than REMC.

**Conclusion:**

The REMC search algorithm used in the original Core Hunter computer program performs well, sometimes leading to slightly better results than some of the simpler methods, although it doesn’t always give the best results. By switching to the new Mixed Replica algorithm overall results and runtimes can be significantly improved. Finally we recommend including minimum distance measures in the objective function when looking for core sets in which all accessions are sufficiently distant from each other. Core Hunter II is freely available as an open source project at http://www.corehunter.org.

## Background

The concept of a core collection was first introduced in [1] and is defined as a representative subset of a given collection sampled with the goal of maximizing diversity and minimizing redundancy. Today, very large germplasm collections numbering hundreds of thousands of accessions of cultivated species and their wild relatives are stored in gene banks throughout the world to preserve crop diversity for further research and application. Due to the size of these collections maintaining all accessions in the active collection and thus in significant quantities to be accessible by researchers and plant breeders is economically prohibitive. Creating core subsets for working collections offers an efficient way of characterizing and utilizing genetic resources without the need of maintaining the entire collection accessible for practical use.

To be able to generate diverse core sets we need evaluation measures that express the diversity of a given collection. These measures are based on a variety of criteria including phenotypic traits or genetic marker data [2-7], or a combination of both [8,9]. Many algorithms for core set selection have been proposed, including stratified sampling techniques. These stratified sampling strategies first perform a clustering of the entire collection and then sample accessions from each cluster, based on some allocation method. The clustering procedure may be based on criteria such as geographical origin, or some phenotypic or genetic distance measure and often hierarchical clustering methods, such as UPGMA [10], are used for this purpose, e.g. in [11-15]. Several allocation methods have been proposed including the P-, L- and D-methods.

The first two of these allocation methods were proposed by Brown in [16] and depend only on the size of the clusters, not on the diversity within each cluster. The P-method and L-method sample accessions from each cluster (i) proportionally to the cluster size and (ii) proportionally to the logarithm of the cluster size, respectively. The D-method was later introduced in [17] as a new allocation method which samples accessions proportionally to the diversity within each cluster so that more diverse clusters contribute more accessions, in order to obtain a high overall diversity in the resulting core. The authors showed that the D-method produced significantly more diverse core collections than other allocation methods.

Another allocation method, the M-method, was proposed by Schoen and Brown in [18]. This method aims at maximizing the number of observed alleles at each marker locus (allelic richness) and determines sample sizes for each cluster according to this objective. The M-method is very useful for preserving rare, low-frequency alleles from the original collection when creating core sets. In fact, this strategy can also be implemented as a non-stratified procedure which samples a core subset directly from the entire collection, optimizing allelic richness. This strategy is employed by the MSTRAT computer program [19] which uses the following steepest descent heuristic algorithm to guide the search: (1) a random initial core set of the desired size *n* is sampled from the entire collection of size *N*, (2) all possible subsets of size *n*−1 of the current core are evaluated and the subset retaining the highest allelic richness score is selected, and (3) from all currently unselected accessions, the accession bringing the highest increase in allelic richness is added to the core, again resulting in a core of size *n*. Steps (2) and (3) are then repeated until no more improvement is observed or until a maximum number of steps have been performed.

It should be noted that the objectives of the D-method and MSTRAT differ. While MSTRAT aims at including rare and localized alleles by maximizing allelic richness, the goal of the D-method is a high representation of the original genetic diversity in the core by including widely adapted accessions that are genetically distant from each other. The former approach is favored by taxonomists and geneticists while the latter corresponds more to the breeder’s preference.

Other non-stratified methods include genetic distance sampling and least distance stepwise sampling. Genetic distance sampling [20] constructs core sets where no two accessions are closer to each other than a given minimum distance threshold, according to some genetic distance measure. This method avoids the need to specify a desired core size, but introduces the threshold as a new input parameter. Least distance stepwise sampling (LDSS) [21] iteratively uses hierarchical clustering to determine which accessions to include or exclude from the core in each search step, until the desired core size has been reached. Both of these methods actively use genetic distances during search, but none of them directly optimizes them.

All of the previously mentioned methods assume that the desired core size (or distance threshold, in case of genetic distance sampling) is known in advance and given as input to the sampling strategy, and then try to create a good core set of the desired size according to the specific objective used. However, a related problem is that of finding the smallest possible core set that retains all unique alleles from the original collection. The PowerCore algorithm was presented in [7] to solve this problem, using a heuristic version of the A∗ shortest path search algorithm. The authors showed that PowerCore was able to find quite small core sets which retained all of the original alleles.

Core Hunter was developed as a new, very flexible framework for selecting core collections [22]. Like MSTRAT, Core Hunter treats core selection as a formal optimization problem by directly sampling from the entire collection, optimizing a given diversity measure. However, Core Hunter uses a more advanced local search technique, Replica Exchange Monte Carlo (REMC) search, to prevent the search stalling in local optima. REMC uses the same criteria as a simulated annealing [23] algorithm for accepting new solutions, except that here several replica solutions are being evaluated at any one time, each with a fixed temperature. Solutions are swapped between neighboring replicas to push the most promising cores towards the coolest replicas, for the sake of convergence. We refer the reader to the original Core Hunter article [22] for a detailed description of this technique. Core Hunter also allows the user to choose which diversity measure will be used for optimization, offering seven widely used genetic evaluation measures including two (mean) genetic distance measures, three diversity indices and two auxiliary measures. Furthermore, it is possible to optimize several measures at once by optimizing a pseudo-index which consists of a linear combination of several of these measures, where the user assigns a weight to each measure according to its importance. In this way one can find core sets with high average genetic distance between accessions and high overall diversity, bringing the breeder’s concept and taxonomist’s perspective closer together. Core Hunter is able to find as good or better core subsets than both MSTRAT and the D-method when optimizing a single measure. Furthermore, when simultaneously optimizing several genetic measures, Core Hunter is often able to construct core sets which simultaneously have higher average genetic distance and overall diversity than any core reported by each of the other two methods. Finally, Core Hunter is able to select smaller core sets than PowerCore that preserve all alleles from the original collection.

In this paper we present Core Hunter II as an extension to the original Core Hunter framework. First we investigate if minimum distance measures, in addition to the available mean distances, ensure that accessions in the core will be sufficiently distant from each other. A second objective is to gain more insight into the performance of the REMC search engine by comparing it with several other heuristic optimization methods implemented in the same flexible Core Hunter framework. In the original Core Hunter article [22], the REMC algorithm was only compared to external methods (MSTRAT, D-method, PowerCore), which were implemented in different frameworks and did not allow the user to choose a specific optimization measure. Implementing several techniques in the same framework allows us to make a fair comparison of the performance of these search techniques. Finally, we assess if the performance of Core Hunter can be further improved in terms of the diversity of selected core sets or the runtime needed to compute them, by switching to a new advanced search technique that uses heterogeneous search replicas: Mixed Replica search (MixRep).

## Methods

We use the same formal definition of the core subset selection problem and multi-objective pseudo-index as in [22]. Here we give only a brief explanation of both concepts. For more details we refer the reader to [22].

### The core subset selection problem

Given some collection *S*, a sampling intensity *γ*, 0 ≤ *γ*≤1 and some diversity measure *F* (possibly a multi-objective pseudo-index), denote *C*(*S*) as the set of all possible subsets of *S* of size *n*=*γ*∗|*S*|. We want to select an optimal^a^ core *c*^∗^∈*C*(*S*) such that *F*(*c*^∗^)=max{*F*(*c*)|*c*∈*C*(*S*)}.

### The multi-objective pseudo-index

Given any *k* measures *F*_*i*_and weights 0≤*a*_*i*_≤1,*i*=1…*k*with

$$\overset{k}{\underset{i=1}{\sum }}a_{i}=1$$

the corresponding pseudo-index is defined as

$$F(c)=a_{1}\cdot F_{1}(c)+a_{2}\cdot F_{2}(c)+\ldots +a_{k}\cdot F_{k}(c)$$

This pseudo-index has no biological meaning at all but just serves as a mean to optimize several measures at once according to their importance (weight).

### Evaluation measures

The original version of Core Hunter only supports genetic marker data (also called molecular marker data) and offers seven diversity measures, including two genetic distance measures, three allelic diversity indices and two auxiliary measures. We briefly discuss these here and refer to [22] and [24] for more details about these and other genetic diversity measures.

Genetic distance measures are defined on pairs of accessions and express their similarity. The higher the distance between two accessions, the more genetically different they are and conversely highly similar accessions can be identified as those being very close to each other. To assess the diversity of an entire collection using a genetic distance measure, it is customary to report the mean distance between all pairs of accessions contained in this collection. These measures are especially useful for breeders who want to ensure that each accession in the selected core set is sufficiently different from the others. Core Hunter offers the Modified Rogers (MR) [2] and Cavalli-Sforza and Edwards (CE) [3] distances which are both Euclidean distances at the allelic level. While MR just treats each allele as a separate dimension using the allelic frequencies as coordinates, CE adopts the square roots of the allelic frequencies as an accession’s coordinates.

The allelic diversity indices are directly computed on the entire core set and are particularly useful for preserving rare alleles, which makes them very well suited for applications aimed at genetic conservation, such as sampling core collections from germplasm resources. Three such diversity indices are available in Core Hunter: (1) Shannon’s diversity index (SH) [4], (2) the expected proportion of heterozygous loci (HE) [5], and (3) the number of effective alleles (NE) [5]. The SH diversity index aims for high allelic diversity in the entire sample, regardless of how different alleles correspond to certain markers, while both HE and NE specifically consider diversity within marker loci.

Finally, two auxiliary measures are also available, expressing the extent to which the original alleles from the entire collection are still present in the core. The allele coverage (CV) [7] simply reports the percentage of preserved alleles and the proportion of non-informative alleles (PN) [6] is defined as the complement of CV measuring the amount of alleles which were completely lost by going from the entire collection to the selected core set. Note that PN is in fact a measure we want to minimize, while maximization is the goal for all six other measures. These auxiliary measures are generally not used as a single objective but as an extra constraint, where the primary goal is optimization of one or more other measures, through the use of a mixed pseudo-index. For precise definitions and formulations of each of these seven measures, we refer to [22] and [24].

### Minimum distance measures

When expressing the diversity of a collection using the mean distance between all pairs of accessions, it is not clear whether optimizing this mean value will in fact lead to cores in which all accessions are sufficiently distant from each other. High mean distance does not *a priori* imply high minimum distance too, so we have extended the Core Hunter framework with two additional distance measures which report the minimum instead of mean distance between all pairs of accessions: (i) minimum Modified Rogers (MRmin) and (ii) minimum Cavalli-Sforza and Edwards (CEmin). We will first assess to what extent optimizing only mean distance leads to high minimum distances and then we will investigate whether it could be beneficial to include these minimum distances in the objective function, either as a replacement for mean distance measures, or in combination with them.

### Performance of REMC

To gain more insight into the performance of the REMC search algorithm that is implemented in the Core Hunter software, we compared its results with those of several simpler methods, implemented in the same flexible Core Hunter framework. All of these are well known basic heuristic search methods: 

1. Standard Local Search (LS) starts with a random solution and then iteratively samples random neighbor solutions, accepting them as the new solution if and only if they are better than the current solution. For our application, we use a so called *single perturbation* neighborhood, which contains all core sets differing in at most one accession from the current core. Possible operations are addition, deletion and swap of one accession.

2. MSTRAT Steepest Descent: as mentioned before, Core Hunter was previously compared with the external MSTRAT program [22], which did not allow the user to choose a specific optimization measure. We have implemented the corresponding steepest descent technique from MSTRAT [19] within the Core Hunter framework for a more fair comparison of the REMC and MSTRAT search engines.

3. LR Greedy Search is a deterministic algorithm that does not take any randomized decision [25]. LR search starts with the empty solution and then iteratively (i) adds the accession that gives rise to the highest increase in diversity, repeated *l* times, (ii) removes an accession while retaining as much of the diversity as possible, repeated *r* times, and (iii) loops back to step (i). The search terminates when the desired core size has been reached. For our experiments, we set *l*=2 and *r*=1, resulting in the deterministic LR(2,1) algorithm^b^ that always performs *n* steps of first adding 2 and then again removing 1 accession, where *n*=|*core*|=*γ*·|*S*|.

For the first two methods and REMC, the stop criteria that decide when to terminate the search^c^ are: (1) maximum runtime – 60 seconds by default, (2) minimum progression, and (3) maximum time without improvement (stuck time). The LR method does not accept such stop criteria as it simply terminates when the desired core size has been reached. For our experiments we set a maximum runtime limit for each randomized algorithm and record both the diversity of the resulting core sets and the corresponding convergence time, which is defined as the point in time from which no more improvement was observed when all figures were rounded to 3 decimal places.

The goal of the comparison is to find out when simple methods break down and where it would be better to turn to more advanced methods such as REMC. These results will give us more insight into the specific characteristics of those problems on which simple methods do or do not fail.

### Mixed Replica Search

In addition to our comparison of REMC with these simpler methods, we also present a new advanced search engine that is inspired by the replicated approach of REMC, but uses heterogeneous instead of homogeneous replicas, which implement different search techniques. We experimented with several simple methods, including those explained in the previous section, and several more advanced heuristics to assess whether we could improve on the results of REMC. We observed that different methods outperformed REMC in several experiments in respect to either the runtime or diversity score, but each method had some drawbacks. Thus, we decided to design one robust Mixed Replica Search engine (MixRep) which combines the strength of several search techniques, to be able to tackle different problems with different techniques without the need of determining in advance which technique is more suited to a specific problem.

The MixRep algorithm is based on four different types of replicas, consisting of two simple and two more advanced search techniques: 

1. LR Semi Replica (LR): a modified version of the deterministic LR(2,1) search avoiding the overhead of exhaustively sampling the first pair of accessions. This replica starts with two randomly chosen accessions and thus introduces a small random effect making the resulting technique no longer purely deterministic.

2. Local Search Replica (LS): this replica performs standard local search using the previously described single perturbation neighborhood.

3. Tabu Search Replica (Tabu): a more advanced technique, based on steepest descent, which always continues with the best neighbor of the current solution even if it is worse than the current solution, but skipping those neighbors that have been declared tabu. In theory all previously visited solutions should be declared tabu. This technique prevents the search from continuously revisiting previous solutions and from traversing cycles within the search space. Our implementation of tabu search uses the steepest descent technique from MSTRAT to construct neighbors.

4. Simple Monte Carlo Replica (MC): these replicas are exactly the same as those used in the REMC search algorithm [22], except that solutions are not exchanged between replicas as the search progresses and temperatures are chosen that are rather low because these advanced replicas are only used in areas containing solutions that are already quite promising^d^.

The algorithm uses only one single LR replica as this method is deterministic (apart from the random selection of the first pair of accessions) so its results show little to no variation. The other three replicas are used repeatedly with different initial solutions. The search process can be described as follows: 

1. One LR replica is created, initialized with a random pair of accessions and activated to run in the background until its search process is complete.

2. Several LS replicas are created and randomly initialized with core sets of the desired size.

3. Until some stop criterion is met, consecutive search rounds are performed containing the following steps: 

(a) All replicas perform some search steps, independently of the other replicas.

(b) The best solution over all replicas is tracked and improvements are reported.

(c) Regularly, new advanced replicas are created (Tabu, MC) and initialized with new cores, obtained by merging promising solutions from the current replicas.

(d) Replicas which did not improve on their current solution during their last couple of search steps are considered to be stuck and subsequently removed.

(e) If the global improvement drops below a certain threshold or if there was no improvement at all for some time, the search is boosted by adding several new randomly initialized LS replicas to provide new variation.

Note that in step (3a) replicas perform their search steps independently from each other, which is in fact also the case in the replicated REMC algorithm [22]. However in the original Core Hunter implementation of REMC the different replicas performed their steps sequentially without taking advantage of this independency. We developed an implementation of step (3a) of the MixRep algorithm that allows the different replicas to be run in parallel in order to achieve an additional improvement in runtimes when computing core sets on machines that have multiple cores and/or processors. In step (3c) initial solutions for the new replicas are created by selecting two promising *parent* solutions from the current replicas and randomly merging these together, an idea inspired by genetic algorithms [26] combining several current solutions into new *children*.

For specific details concerning the MC replicas we refer to [22] as these are the same as those used by REMC, except here they are set with rather low temperatures. The Tabu replicas perform a modified version of the theoretic tabu ideology that was explained before. In practice declaring all previously visited solutions tabu both requires (i) a lot of memory to store the history containing all these solutions and (ii) many computations to check whether some new solution is already present in the history and therefore tabu. This check is especially complex and time consuming because every solution is in fact a set of accessions that needs to be compared for equality and not just a singe accession. Therefore, a limited history (also called tabu list) is maintained that only remembers the most recent solutions and forgets everything that happened before. The scope of the history is controlled by the tabu list size which defines how many previous steps are remembered. Even then, storing and comparing entire core sets in this history is highly impractical. Therefore, instead of declaring the exact solutions tabu, we declare some specific actions tabu, which are well chosen to prevent returning to these previously visited solutions^e^. When the current solution has been perturbed into one of its neighbors by changing the accession at index *i*, changes at this index are no longer allowed as long as the tabu list contains the index *i*. This way we only need to store a list of integers, which uses less memory and allows for fast comparison, and still fulfills the requirement that previously visited solutions are defined as tabu. However, this method also declares some other solutions tabu, which may not yet have been visited at all, making this approach too restrictive and possibly prohibiting some very promising solutions. Therefore, we add an *aspiration criterion* that overrides the tabu in case of a neighbor having a higher score than the currently-known best solution. In this case such solution is clearly only tabu due to the index approach and cannot have been visited before. It has to be noted however that this is still more restrictive than storing all previously visited solutions in a tabu list.

In summary, the Mixed Replica algorithm starts with some simple, fast methods to perform an initial exploration of the search space. Afterwards, more advanced methods take over, starting in these areas where the simple methods had arrived. On average these areas of the search space contain generally better solutions, thus presenting a more difficult task of further improving on the current solution. As soon as little or no more improvement is being made the search is boosted by introducing new simple, fast methods, starting from new random points in the search space to supply new variation. The best solution over all replicas is tracked at all times and reported when some stop criterion is met, by default after a maximum total runtime of 60 seconds.

### Datasets

We performed intensive experiments using five different realistic datasets, including the larger two datasets used in the original Core Hunter article [22]: the bulk [27] and accession [28] maize datasets. In addition to these maize datasets we also performed experiments for three larger sets including one flax [29] and two pea [30-32] datasets. Details concerning these five datasets are given below: 

● ‘bulk maize data set’ [27]: 

  – 275 samples, genotyped at 24 SSR loci with 186 total alleles

  – obtained by fingerprinting 275 bulks of maize landrace populations, each containing multiple maize individuals from the Americas and Europe using 24 multi-allelic SSR markers

● ‘accession maize data set’ [28]: 

  – 521 samples, genotyped at 26 SSR loci with 209 total alleles

  – obtained by fingerprinting 521 maize individuals from 25 different populations using 26 multi-allelic SSR markers

● ‘flax data set’ [29]: 

  – 708 samples, genotyped at 141 IRAP loci with 282 total ‘alleles’

  – obtained by fingerprinting 708 bulks of 10 flax individuals each using 141 IRAP markers (similar to AFLP); only two possible states occur for each bulk at each marker locus: (i) presence of allele and (ii) absence or marker failure, where it is not possible to distinguish between these last two states

● ‘pea data set’ [30]: 

  – 1283 samples, genotyped at 19 RBIP loci with 38 total ‘alleles’

  – obtained by fingerprinting 1283 bulks of 10 pea individuals each using 19 RBIP markers, with 4 different possible states for each bulk at each maker locus: (i) presence of allele in each individual, (ii) absence in each individual, (iii) mixed state having both individuals with presence and absence in the same bulk, and finally (iv) the zero state which means no data is available

● ‘large pea data set’ [31,32]: 

  – 4429 samples, genotyped at 17 RBIP loci with 34 total ‘alleles’

  – obtained in the same setting as the previous dataset, but containing significantly more samples (again bulks of 10 individuals)

### Implementation and hardware

Extensions to the original Core Hunter software were implemented in Java (version 1.6), starting from the original code which was kindly provided by the authors. All of our main experiments were performed on a 2.53 GHz Intel Core i5 dual core MacBook Pro with 4 GB of RAM and 256 KB of CPU cache per core. Some additional experiments were run on the UGent ‘helios’ computing server, a 2 × 6 core machine which has two 6-core 3.07 GHz Intel Xeon X5675 processors, 48 GB of RAM and 12 MB cache for each CPU, running Debian Linux. We will explicitly note which experiments were run on this helios server.

The statistical R software was used to produce all visualizations of datasets and sampled cores. Principal component analyses were performed using the built-in R command prcomp.

## Results and discussion

First we will present results of a comparison of REMC with the more simple methods described in the previous section, using the original Core Hunter evaluation measures. Then we will illustrate a possible problem regarding minimum distances if mean distances are optimized alone, using some generated toy example datasets of low dimension^f^. Next, the impact of including these newly introduced minimum distances in the objective function when sampling from the realistic datasets will be discussed.

Based on these results, we will give further motivation of the specific composition of our new Mixed Replica algorithm and then we will discuss this method’s performance regarding both the diversity of the constructed core sets and the runtimes until convergence. To investigate the impact of the sampling intensity on the performance of our algorithms, all experiments have been repeated for two different sampling intensities (int): 20% (fairly large) and 5% (rather low), both within the range of sampling intensities proposed in previous research [16,18].

### Performance of REMC using original measures

Table 1 shows the results of comparing REMC with the Local Search, MSTRAT and LR(2,1) algorithms described above, with a large sampling intensity of 20% and using the original Core Hunter diversity measures. For most combinations of algorithm, dataset and evaluation measure, we applied the default maximum runtime of 60 seconds as the only stop criterion^g^. Only for the large pea dataset was the runtime limit set to 10 minutes due to its large size. No runtime limit holds for LR search. Both the diversity scores of the constructed core sets and their corresponding convergence times (smaller figures) are presented. The latter is defined as the point in time from which no more improvement was observed^h^. In cases where several methods gave different results in terms of the reported core diversity, the highest score is shown in bold. For each dataset the bottom line shows the corresponding diversity scores of the entire collection to allow comparison with the scores of the selected cores. Single measure optimizations were performed for each of the available (mean) distance measures (MR, CE) and diversity indices (SH, HE, NE), but not for the auxiliary measures (PN, CV). In practice PN and CV are not generally used as a single objective but as additional constraints when the main goal is optimization of one or more of the other measures. The mixed pseudo-index does contain all seven measures with equal weights, including these auxiliary measures.

**Table 1:** Comparison of REMC with simpler methods – original measures (int = 0.2)

Algorithm^*^	**MR**	**(t)**	**CE**	**(t)**	**SH**	**(t)**	**HE**	**(t)**	**NE**	**(t)**	Mixed^**^	**(t)**
	**Bulk maize data set (275)**											
Local S.	0.572	0.45s	0.641	0.55s	4.531	0.35s	0.667	0.25s	3.446	0.65s	**10.680**	15.0s
MSTRAT	0.572	0.31s	0.641	0.32s	4.531	0.38s	0.667	0.43s	3.446	0.43s	10.678	1.5s
LR(2,1)	0.572	0.61s	0.641	0.64s	4.531	1.1s	0.667	1.0s	3.446	1.0s	**10.680**	4.2s
REMC	0.572	1.0s	0.641	2.0s	4.531	2.0s	0.667	1.0s	3.446	3.0s	**10.680**	15.0s
Original	0.440		0.521		4.399		0.620		2.937			
	**Accession maize data set (521)**											
Local S.	**0.695**	2.0s	0.752	1.0s	4.670	1.0s	0.676	0.45s	3.501	2.0s	11.086	15.0s
MSTRAT	**0.695**	1.7s	0.752	1.7s	4.670	1.6s	0.676	1.5s	3.501	1.5s	11.083	8.2s
LR(2,1)	**0.695**	2.9s	0.752	2.9s	4.670	4.2s	0.676	3.9s	3.502	3.9s	**11.087**	17.5s
REMC	0.694	4.0s	0.752	4.0s	4.670	5.0s	0.676	3.0s	3.502	20.0s	11.086	50.1s
Original	0.630		0.696		4.467		0.591		2.742			
	**Flax data set (708)**											
Local S.	**0.512**	2.1s	**0.512**	2.1s	5.340	0.58s	**0.263**	0.58s	1.469	1.1s	**8.878**	12.7s
MSTRAT	**0.512**	5.1s	**0.512**	5.1s	5.340	3.7s	**0.263**	3.8s	1.469	3.8s	8.877	25.1s
LR(2,1)	**0.512**	7.4s	**0.512**	7.4s	5.340	13.3s	**0.263**	12.9s	1.469	12.8s	**8.878**	50.4s
REMC	0.511	5.0s	0.511	4.0s	5.340	30.0s	0.262	4.0s	1.469	30.0s	8.874	60.4s
Original	0.468		0.468		5.285		0.222		1.377			
	**Pea data set (1283)**											
Local S.	**0.593**	3.0s	**0.597**	2.7s	**3.556**	1.1s	**0.440**	1.0s	**1.867**	6.3s	**7.946**	53.6s
MSTRAT	**0.593**	28.8s	**0.597**	28.5s	**3.556**	17.5s	**0.440**	18.3s	**1.867**	18.2s	7.851	60.6s
LR(2,1)	**0.593**	34.1s	**0.597**	34.3s	**3.556**	24.5s	**0.440**	28.3s	**1.867**	27.9s	**7.946**	03m03s
REMC	0.591	50.0s	0.595	30.0s	3.553	7.0s	0.437	15.0s	1.865	15.0s	7.876	61.2s
Original	0.509		0.515		3.482		0.381		1.713			
	**Large pea data set^▾^ (4429)**											
Local S.	**0.594**	49.4s	**0.596**	38.1s	**3.486**	18.3s	**0.465**	16.9s	**1.886**	23.8s	**7.947**	07m43s
MSTRAT	0.555	10m03s	0.558	10m03s	3.478	10m03s	0.458	10m03s	1.866	10m02s	7.396	10m07s
LR(2,1)	**0.594**	42m56s	**0.596**	42m35s	**3.486**	21m18s	**0.465**	21m24s	**1.886**	21m23s	**7.947**	04h08m
REMC	0.577	03m41s	0.580	08m49s	3.470	08m37s	0.448	04m29s	1.875	05m22s	7.621	10m03s
Original	0.464		0.466		3.348		0.352		1.609			

^*^For each combination of algorithm, dataset and evaluation measure, 20 independent runs were performed from which averaged results are reported. By default runs were limited by a runtime of 60 seconds, except for the large pea dataset where a runtime limit of 10 minutes was applied. Furthermore the LR method does not accept a runtime limit but continues search until the desired core size has been reached.

^**^Results shown are those of a pseudo-index containing all seven measures with equal weights.

^▾^These results were computed on the helios server.

As we can see, results are very similar for each of the four algorithms. The advanced REMC algorithm never outperforms all of the simple methods when comparing the diversity scores of the constructed core sets for a specific dataset and evaluation measure. More accurately, REMC never outperforms LR search and only occasionally presents slightly better results than Local Search and/or MSTRAT. Except for the smallest (bulk) maize dataset, some or even all simple methods often slightly outperform REMC. However differences in diversity are never significant. The largest difference is observed when optimizing the mixed objective function for the large pea dataset, where both Local Search and LR outperform REMC with a relative improvement of about 4%. It should be noted that in this case LR takes much more time than the runtime limit imposed on the other methods.

For the large pea dataset in general both REMC and MSTRAT result in somewhat worse scores than Local Search and LR. Furthermore simple Local Search is much faster than any other method including REMC, with convergence times below one minute for each single measure and of about 7 minutes in case of a mixed objective. Although LR reaches very similar or the same scores as Local Search for this large dataset, it is a lot slower with runtimes up to several hours. This longer runtime is due to the fact that LR starts with an empty solution and has to perform a fixed number of steps relative to the core size, which depends on the original dataset size and given sampling intensity. For large datasets and intensities, this process becomes slower and for the evaluation measures used it clearly does not offer any gain in diversity compared with the very fast Local Search. A similar speed issue also applies for MSTRAT, as this method evaluates many neighbors in each step, again relative to the dataset size. Furthermore, MSTRAT sometimes results in lower scores than Local Search, for example in the case when analyzing the large pea dataset.

For the smaller datasets runtimes of Local Search are also often significantly lower than those of the advanced REMC method, which is not surprising since REMC performs computations for several search replicas. It is mainly due to this reduced runtime that Local Search is sometimes able to construct slightly more diverse core sets than REMC, within the imposed time limit. By performing some informal experiments with higher runtime limits, we learned that in most cases REMC finds these results too when given more time.

Table 2 presents the same results, but now for a lower sampling intensity of only 5%. Results are similar although for the smaller datasets differences in diversity scores are now somewhat higher and here REMC sometimes just outperforms each of the simple methods, e.g. when optimizing the SH or NE measure for the bulk maize dataset or the CE, HE, NE or mixed measure for the accession maize dataset. However, differences in diversity are again not significant in any case and Local Search is much faster than the other algorithms.

**Table 2:** Comparison of REMC with simpler methods – original measures (int = 0.05)

Algorithm^*^	**MR**	**(t)**	**CE**	**(t)**	**SH**	**(t)**	**HE**	**(t)**	**NE**	**(t)**	Mixed^**^	**(t)**
	**Bulk maize data set (275)**											
Local S.	0.643	0.25s	**0.700**	0.25s	4.567	0.25s	0.685	0.25s	3.625	0.35s	10.781	0.9s
MSTRAT	0.643	0.14s	0.699	0.14s	4.567	0.15s	0.685	0.15s	3.616	0.15s	10.772	0.46s
LR(2,1)	0.643	0.34s	**0.700**	0.37s	4.565	0.68s	0.685	0.62s	3.605	0.59s	**10.790**	2.2s
REMC	0.643	0.35s	**0.700**	0.35s	**4.568**	0.65s	0.685	0.55s	**3.631**	3.0s	**10.790**	7.0s
	0.440		0.521		4.399		0.620		2.937			
	**Accession maize data set (521)**											
Local S.	**0.723**	0.45s	0.781	0.35s	**4.724**	0.95s	0.701	0.35s	3.880	2.0s	11.210	4.0s
MSTRAT	0.722	0.26s	0.781	0.27s	4.723	0.34s	0.701	0.32s	3.874	0.32s	11.200	1.1s
LR(2,1)	**0.723**	1.2s	0.781	1.2s	**4.724**	2.1s	0.701	2.1s	3.861	2.1s	11.206	6.8s
REMC	**0.723**	0.75s	**0.782**	2.0s	**4.724**	2.0s	**0.702**	5.0s	**3.886**	6.0s	**11.216**	50.0s
Original	0.630		0.696		4.467		0.591		2.742			
	**Flax data set (708)**											
Local S.	0.533	1.0s	0.533	1.0s	5.358	0.60s	0.278	0.69s	1.504	1.2s	**8.965**	12.8s
MSTRAT	0.533	0.66s	0.533	0.67s	5.358	0.73s	0.278	1.1s	1.504	1.1s	8.960	3.5s
LR(2,1)	0.533	3.3s	0.533	3.3s	5.358	7.3s	0.278	7.0s	1.504	7.0s	8.962	22.0s
REMC	0.533	3.0s	0.533	3.0s	5.358	2.0s	0.278	3.0s	1.504	4.0s	**8.965**	30.0s
Original	0.468		0.468		5.285		0.222		1.377			
	**Pea data set (1283)**											
Local S.	0.626	1.4s	0.629	1.2s	3.578	0.50s	0.451	0.40s	1.898	0.70s	**8.084**	33.1s
MSTRAT	0.626	1.5s	0.629	1.6s	3.578	1.1s	0.451	1.0s	1.898	1.0s	8.083	6.9s
LR(2,1)	0.626	3.5s	0.629	3.5s	3.578	4.8s	0.451	4.8s	1.898	4.8s	8.083	18.3s
REMC	0.626	10.0s	0.629	7.0s	3.578	2.0s	0.451	3.0s	1.898	2.0s	8.083	40.0s
Original	0.509		0.515		3.482		0.381		1.713			
	**Large pea data set^▾^ (4429)**											
Local S.	**0.635**	09.4s	**0.637**	23.9s	**3.518**	02.4s	**0.496**	15.1s	**1.983**	27.5s	**8.158**	04m17s
MSTRAT	**0.635**	49.6s	**0.637**	49.6s	**3.518**	24.5s	**0.496**	25.4s	**1.983**	25.5s	**8.158**	03m44s
LR(2,1)	**0.635**	01m18s	**0.637**	01m19s	**3.518**	01m05s	0.495	01m18s	1.981	01m09s	**8.158**	07m42s
REMC	0.633	06m05s	0.634	36.8s	3.515	27.6s	0.492	13.9s	1.982	06m53s	8.147	09m36s
Original	0.464		0.466		3.348		0.352		1.609			

^*^For each combination of algorithm, dataset and evaluation measure, 20 independent runs were performed from which averaged results are reported. By default runs were limited by a runtime of 60 seconds, except for the large pea dataset where a runtime limit of 10 minutes was applied. Furthermore the LR method does not accept a runtime limit but continues search until the desired core size has been reached.

^**^Results shown are those of a pseudo-index containing all seven measures with equal weights.

^▾^These results were computed on the helios server.

We conclude that for the original Core Hunter evaluation measures, simple methods perform very well. In most of our experiments at least one and often all simple methods were able to construct equal or slightly more diverse core sets than REMC, while their runtimes are often significantly lower, especially those of simple Local Search. Runtimes of both LR and MSTRAT are very sensitive to the dataset and core size, so these methods become slower for large datasets and intensities. In case of fairly low sampling intensities it is harder for the simple methods to create good core sets and in this case they are sometimes just outperformed by REMC, but differences are never significant. So in conclusion for these measures simple methods present very similar results while often using less computation time. Simple Local Search is clearly the fastest of all considered methods and often it also gives the best results. It should be noted however that when comparing the results of the simple methods, it is not always the same method that outperforms all the others.

### Minimum distance measures

**Figure 1:**
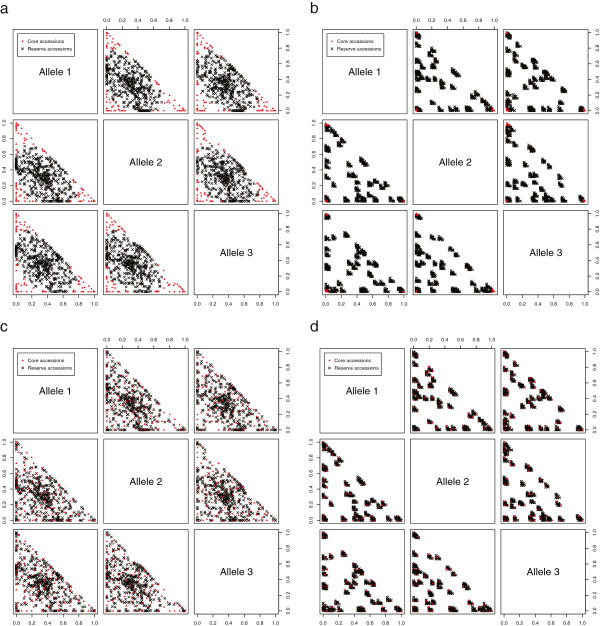
**3D Toy example datasets, optimizing mean versus minimum distance.** Core collections sampled from two generated three-dimensional toy example datasets, respectively of size 500 and 1000, the former being completely random, the latter having a very strongly clustered structure. Both datasets contain only one single marker with 3 corresponding alleles. Core selection was performed using the REMC algorithm, optimizing mean (top) and minimum (bottom) MR distances. For the random dataset, the sampling intensity is set to 0.2, while an intensity of 0.05 is used for the larger, clustered set. **(a)** random dataset, mean Modified Rogers’ distance (sampling intensity = 0.2), **(b)** clustered dataset, mean Modified Rogers’ distance (sampling intensity = 0.05), **(c)** random dataset, minimum Modified Rogers’ distance (sampling intensity = 0.2), **(d)** clustered dataset, minimum Modified Rogers’ distance (sampling intensity = 0.05).

Figure 1 shows two generated three-dimensional datasets, respectively of size 500 (left) and 1000 (right), where all accessions of the former dataset are created completely at random while the latter set is strongly clustered. Both datasets have only one single marker with 3 corresponding alleles. For the random dataset allelic frequencies for each accession were randomly set with a uniformly distributed value between 0.0 and 1.0 followed by normalization so that the sum of the three frequencies equals 1. The clustered dataset was created in a similar way, where first 50 random accessions were picked to serve as cluster centers and then other accessions were added by repeatedly perturbing these centers to create clusters of accessions.

When sampling core collections from these datasets with the objective of optimizing mean MR distance (MR), REMC constructed the core sets displayed in Figure 1(a) and 1(b). It is clear that both of these cores have a very low minimum distance and that they are not at all representative of the structure of the original collections, although high mean distances have indeed been obtained. The selected core shown in Figure 1(b), which is sampled from the clustered dataset, simply consists of several very dense extreme clusters but to create a representative core we should in fact sample accessions from many different clusters. Optimizing only the mean distance seems to select accessions near extremes along the various dimensions, which indeed leads to high mean scores. In our examples this effect results in sampling some very similar or even *identical* accessions, where the latter are defined as accessions for which all available allelic frequencies are equal. One possible problem is that selecting extremes is not sufficient to reach high minimum distances when the size of the sampled core is significantly larger than the dimension of the dataset. In addition to selecting extremes, we should sample many accessions in between the extremes to keep them sufficiently distant from each other. Although both of these toy examples are clearly extreme cases, datasets with many accessions and relatively few total alleles do in fact also occur in practice (e.g. our pea datasets) and therefore we should be careful when only optimizing mean distances. Furthermore, many possible core subsets have highly similar mean distances and it might very well be possible to select one which does have high minimum distance, while still retaining high mean distance.

Figures 1(c) and 1(d) display core sets that were constructed by REMC optimizing minimum instead of mean MR distance (MRmin). Both of these cores are more representative for the original collection than those obtained by optimizing mean distance. The core displayed in Figure 1(d) clearly contains accessions from many or even all different clusters instead of just some extreme clusters. By optimizing minimum distance the extremes are still included in the cores, but many accessions in between these extremes were also sampled. These results indicate that it is useful to include minimum distances in the objective function, possibly replacing mean distances or in combination with them, to create cores with high distance between each pair of accessions.

### Performance of REMC using minimum distances

Now we will present results of using these new minimum distance measures when sampling from realistic datasets. Table 3 presents results of optimizing minimum versus mean MR distances with a sampling intensity of 20%, for REMC and the three simple methods. For each combination of dataset and algorithm, three different experiments were performed: (i) optimizing mean MR alone (MR), (ii) optimizing minimum MR alone (MRmin), and (iii) optimizing a mixed objective which contains both, with an equal weight of 0.5 (MixedMR). For the first two cases, averages of the other measure’s score (respectively MRmin and MR) are also shown, which were computed afterwards on the constructed cores and were not used during optimization. In case of the mixed objective, the MRmin and MR components are presented so we can compare both the mean and minimum distances of these cores with those constructed in other experiments.

**Table 3:** Comparison of REMC with simpler methods – minimum versus mean MR (int = 0.2)

**Optimized →**	**MR**					**MRmin**					MixedMR^**^			
Algorithm^*^	**MR**	**(t)**	**MRmin**^**∙**^			**MRmin**	**(t)**	**MR**^**∙**^			**MixedMR**	**(t)**	**MRmin**^**∘**^	**MR**^**∘**^
						**Bulk maize data set (275)**								
Local S.	0.572	0.45s	0.258			0.392	4.9s	0.548			0.471	2.6s	0.380	**0.561**
MSTRAT	0.572	0.31s	0.258			0.386	1.8s	0.543			0.470	1.2s	0.380	0.560
LR(2,1)	0.572	0.61s	0.258			0.393	1.2s	0.549			0.473	1.5s	0.393	0.553
REMC	0.572	1.0s	0.258			**0.397**	35.6s	0.549			**0.476**	23.6s	**0.395**	0.557
Original	0.440		0.116			0.116		0.440					0.116	0.440
						**Accession maize data set (521)**								
Local S.	**0.695**	2.0s	0.392			0.404	0.40s	0.630			0.582	4.3s	0.471	**0.694**
MSTRAT	**0.695**	1.7s	0.392			0.403	0.32s	0.631			0.583	4.1s	0.473	**0.694**
LR(2,1)	**0.695**	2.9s	0.392			**0.555**	4.3s	0.670			**0.618**	5.9s	**0.555**	0.681
REMC	0.694	4.0s	0.392			0.497	56.7s	0.646			0.608	51.0s	0.526	0.690
Original	0.630		0.294			0.294		0.630					0.294	0.630
						**Flax data set (708)**								
Local S.	**0.512**	2.1s	0.223			0.226	0.60s	0.468			0.406	6.4s	0.300	**0.512**
MSTRAT	**0.512**	5.1s	0.223			0.226	1.2s	0.469			0.404	12.5s	0.296	**0.512**
LR(2,1)	**0.512**	7.4s	0.223			**0.377**	10.6s	0.494			**0.443**	15.7s	**0.386**	0.499
REMC	0.511	5.0s	0.213			0.315	30.9s	0.475			0.422	39.5s	0.337	0.508
Original	0.468		0.000			0.000		0.468					0.000	0.468
						**Pea data set (1283)**								
Local S.	**0.593**	3.0s	0.000			0.000	0.10s	0.509			0.302	4.6s	0.011	**0.593**
MSTRAT	**0.593**	28.8s	0.000			0.000	0.63s	0.510			0.299	60.7s	0.006	0.592
LR(2,1)	**0.593**	34.1s	0.000			**0.324**	50.2s	0.569			**0.454**	01m15s	**0.324**	0.583
REMC	0.591	50.0s	0.000			0.006	36.6s	0.510			0.375	60.4s	0.166	0.583
Original	0.509		0.000			0.000		0.509					0.000	0.509
						**Large pea data set^▾^ (4429)**								
LR(2,1)	**0.594**	42m56s	0.000			**0.243**	52m46s	0.554			**0.411**	01h35m	**0.243**	**0.579**
REMC	0.577	03m41s	0.000			0.000	0.19s	0.463			0.273	09m08s	0.000	0.546
Original	0.464		0.000			0.000		0.464					0.000	0.464

^*^For each combination of algorithm, dataset and evaluation measure, 20 independent runs were performed from which averaged results are reported. By default runs were limited by a runtime of 60 seconds, except for the large pea dataset where a runtime limit of 10 minutes was applied. Furthermore the LR method does not accept a runtime limit but continues search until the desired core size has been reached.

^**^Results shown are those of a pseudo-index containing both minimum and mean MR distance, with equal weight = 0.5.

^∙^Not used during optimization, but computed afterwards on the constructed core sets.

^∘^Components of mixed MR measure.

^▾^These results were computed on the helios server.

The results show that differences between diversity scores reported by the different algorithms are generally much bigger here than for the original Core Hunter measures discussed before. Local Search and MSTRAT perform worse than LR and REMC in all experiments using either MRmin or the mixed MR objective. The differences are more obvious for the larger datasets where it is more difficult to obtain high minimum distances because more accessions have to be selected. In this case both Local Search and MSTRAT break down when minimum distances are included in the objective. As this effect was already clearly visible from the results of the first four datasets, Local Search and MSTRAT were not used for the large pea dataset experiments. Interestingly it is not the advanced REMC, but LR which leads to the highest minimum distances for the larger datasets. Only for the smallest dataset (bulk maize) does REMC outperform LR and differences between their results increase for larger datasets.

When using mean MR alone, sampling from both pea sets leads to a minimum distance of zero for all algorithms, which means that accessions that are identical^i^ have been selected. In fact these datasets suffer from the same problem as the toy examples presented before, having many accessions and only few total alleles. The size of the selected core (size > 250 for pea set, and > 800 for large pea) is significantly larger than the dimension of the dataset (dim < 40). Optimizing only mean distance is not enough to guarantee high minimum distance and for these sets some identical accessions were selected in the core. Yet, by using the mixed MR objective, the LR method is able to sample cores from the smallest pea dataset with a quite high minimum MR distance of 0.324 while retaining a mean MR of 0.583, only less than 2% lower than the value of 0.593 which was obtained when optimizing mean MR alone. Even for the large pea dataset, LR reports a fairly large minimum distance of 0.243 together with only a small decrease of less than 3% in mean distance score. Note that REMC – even when using the mixed objective – reports much lower minimum distances for these pea sets. For the large pea dataset REMC still samples cores with zero minimum distance. These results suggest that much higher minimum distances can be reached, while retaining similar mean distances, by including both measures in the objective function and using a well chosen, suitable algorithm.

For the smaller datasets, this same conclusion holds. Although these datasets don’t suffer from the dimensionality problem and already reach acceptable minimum distances with mean MR alone, using the mixed MR objective still results in higher minimum distances while retaining most of the mean score, compared to mean MR alone. Across all experiments, gains in minimum distance range from 20.03% to 73.21% (and in fact infinite relative improvement for the pea datasets), while losses in mean are always smaller than 5.36%. For these datasets therefore it might also be useful to include minimum distances in the objective.

Optimizing minimum distance alone however would not be a good idea, because this presents two problems originating from the fact that many sets have exactly the same minimum MR distance. First, some of these sets might very well have higher mean values than others and we want to favor these. Second, having many possible cores with equal score makes finding a good solution more difficult to solve with optimization algorithms. This effect can be noticed in our results, as the obtained minimum distance values are often higher when optimizing the mixed MR objective compared to optimizing minimum distance alone. Minimum distances should therefore be used as additional constraints by including them in the objective without leaving out the original mean distance measures.

Finally it should again be noted that although LR seems to be very well suited for optimization of minimum distances this method becomes slower for large datasets. This problem with LR is most obvious in the case of large datasets and intensities leading to large core sizes, because of its deterministic nature, starting with an empty solution. For the large pea dataset LR requires much more time than the runtime limit applied to REMC. Because of this big difference in runtimes for the large pea dataset we experimented with applying higher runtime limits to REMC, but even when going up to a limit of 2 hours instead of 10 minutes results of REMC almost do not improve compared to the results shown in Table 3, and REMC still does not succeed in sampling cores with non-zero minimum distance (results not shown). So it is clear that LR is indeed more suited for optimizing minimum distances than any of the other methods. However if minimum distances are not important the LR method should not be used in favor of simple Local Search, as then Local Search samples similar cores quicker. Only when high minimum distances are required it might be worth running LR and waiting somewhat longer for the results.

**Figure 2:**
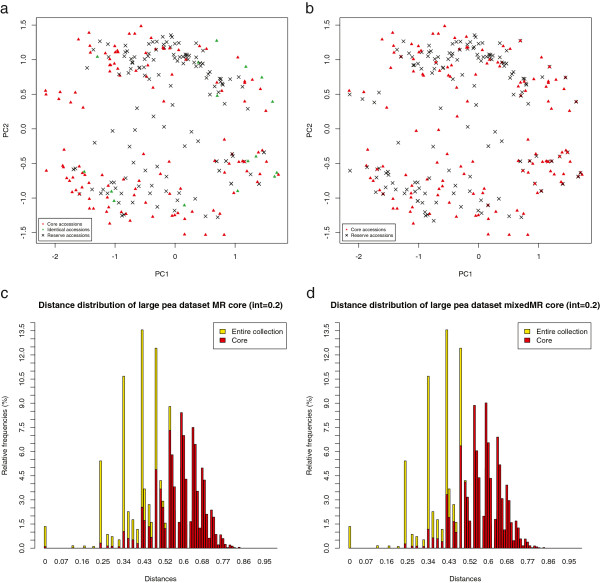
**PCA plots and distance histograms of cores sampled from large pea dataset.** This figure shows both PCA plots and distance histograms of core collections sampled from the large pea dataset, once obtained by optimizing mean MR alone and once by optimizing the mixed MR objective which includes both mean and minimum MR distance with equal weight. The sampling intensity was set to 0.2 and cores where constructed using the LR method. **(a)** optimizing mean Modified Rogers’ distance – core structure, **(b)** optimizing mixed Modified Rogers’ distance – core structure, **(c)** optimizing mean Modified Rogers’ distance – pairwise distance distribution, **(d)** optimizing mixed Modified Rogers’ distance – pairwise distance distribution.

To investigate the results in greater detail we performed a principal component analysis (PCA) of several core sets selected from the large pea dataset and compared the distribution of the distances between these selected accessions and those of the entire collection. Figure 2 presents PCA plots of cores (and their corresponding distance histograms) selected by the LR method optimizing mean MR alone (Figure 2(a) and 2(c)) and the mixed MR objective (Figure 2(b) and 2(d)) with a sampling intensity of 20%. The core plots only show the first two principal components for clarity. However, some of the additional components still showed significant variance after PCA analysis, so we filtered both the original dataset and selected cores by removing all accessions which had extreme values in at least one of these dropped components, where extreme means beyond the inner 75% of its total range, to get a clear view of the structure of the selected cores regarding only these first two principal components.

These plots indicate that both the selected core accessions and their corresponding distances are quite similar for both objective functions, with two important differences. First, the core plots clearly show that when optimizing mean MR alone, several identical accessions are selected (green rectangles), while none of these are selected using the mixed distance measure. Second, using mixed MR leads to the selection of more intermediate accessions, while mean MR leaves more of a gap near to the center of the space. Similar differences can be observed from the corresponding distance distributions. The histogram in Figure 2(c) (MR) shows a small peak at the zero distance indicating some identical accessions are present inside the core. Even though relatively few identical accessions were selected with respect to the total core size, these are still of no practical use and should be avoided if possible. Figure 2(d) again shows that these identical accessions have been successfully avoided by using the mixed objective and in return some more intermediate accessions were selected as indicated by the slightly higher peaks near the mean of the distribution. Besides these small but yet important differences, both distributions are similar which explains the similar mean distance scores of both approaches. Furthermore, the mean of both distributions has been successfully shifted to a significantly larger value than that of the original distribution, shown in the background.

Table 4 shows results using the same settings but now for a smaller sampling intensity of only 5%, leading to the same conclusions as those seen for 20%. With these smaller core subsets non-zero minimum distances are obtained for the smallest pea dataset when using mean MR, but not for the large pea dataset. Including minimum distances in the objective again boosts the minimum scores of these cores, while retaining high mean distances. Furthermore, with this smaller sampling intensity LR does not outperform REMC for the maize and flax datasets, but it still does for the larger pea datasets. This result clearly shows that the advantage of LR compared to REMC depends on the core size and is only present for larger cores. However for smaller cores, results of both methods are very similar. Similar results were also found for cores generated using the mean and minimum CE distance measures. These results can be found in (see Additional file 1: Tables S1 and S2).

**Table 4:** Comparison of REMC with simpler methods – minimum versus mean MR (int = 0.05)

**Optimized →**	**MR**					**MRmin**					MixedMR^**^			
Algorithm^*^	**MR**	**(t)**	**MRmin**^**∙**^			**MRmin**	**(t)**	**MR**^**∙**^			**MixedMR**	**(t)**	**MRmin**^**∘**^	**MR**^**∘**^
						**Bulk maize data set (275)**								
Local S.	0.643	0.25s	0.438			0.529	0.60s	0.613			0.578	0.50s	0.516	0.641
MSTRAT	0.643	0.14s	0.353			0.522	0.42s	0.608			0.578	0.31s	0.513	**0.643**
LR(2,1)	0.643	0.34s	0.513			0.534	0.52s	0.622			0.576	0.72s	0.523	0.628
REMC	0.643	0.35s	0.513			**0.539**	1.8s	0.615			**0.582**	4.7s	**0.534**	0.629
Original	0.440		0.116			0.116		0.440					0.116	0.440
						**Accession maize data set (521)**								
Local S.	**0.723**	0.45s	0.511			0.495	0.10s	0.634			0.663	1.5s	0.607	0.719
MSTRAT	0.722	0.26s	0.476			0.490	0.17s	0.635			0.651	0.59s	0.581	**0.721**
LR(2,1)	**0.723**	1.2s	0.510			0.620	1.8s	0.699			0.674	2.3s	0.635	0.712
REMC	**0.723**	0.75s	0.519			**0.630**	58.7s	0.700			**0.678**	56.0s	**0.638**	0.717
Original	0.630		0.294			0.294		0.630					0.294	0.630
						**Flax data set (708)**								
Local S.	0.533	1.0s	0.337			0.309	0.20s	0.470			0.475	2.6s	0.418	**0.532**
MSTRAT	0.533	0.66s	0.341			0.320	0.36s	0.470			0.468	1.6s	0.405	**0.532**
LR(2,1)	0.533	3.3s	0.357			**0.446**	4.1s	0.515			0.481	6.2s	**0.446**	0.517
REMC	0.533	3.0s	0.337			0.429	44.3s	0.505			**0.487**	20.3s	**0.446**	0.529
Original	0.468		0.000			0.000		0.468					0.000	0.468
						**Pea data set (1283)**								
Local S.	0.626	1.4s	0.200			0.122	0.10s	0.510			0.481	2.7s	0.338	0.624
MSTRAT	0.626	1.5s	0.209			0.104	0.25s	0.510			0.454	4.3s	0.282	**0.625**
LR(2,1)	0.626	3.5s	0.229			**0.429**	5.3s	0.595			**0.520**	7.7s	**0.429**	0.611
REMC	0.626	10.0s	0.246			0.328	60.5s	0.552			0.510	26.1s	0.397	0.622
Original	0.509		0.000			0.000		0.509					0.000	0.509
						**Large pea data set^▾^ (4429)**								
LR(2,1)	**0.635**	01m18s	0.000			**0.343**	01m55s	0.597			**0.488**	03m04s	**0.364**	0.611
REMC	0.633	06m05s	0.000			0.000	0.13s	0.462			0.313	02m03s	0.000	**0.626**
Original	0.464		0.000			0.000		0.464					0.000	0.464

^*^For each combination of algorithm, dataset and evaluation measure, 20 independent runs were performed from which averaged results are reported. By default runs were limited by a runtime of 60 seconds, except for the large pea dataset where a runtime limit of 10 minutes was applied. Furthermore the LR method does not accept a runtime limit but continues search until the desired core size has been reached.

^**^Results shown are those of a pseudo-index containing both minimum and mean MR distance, with equal weight = 0.5.

^∙^Not used during optimization, but computed afterwards on the constructed core sets.

^∘^Components of mixed MR measure.

^▾^These results were computed on the helios server.

We conclude that Local Search is no longer the most promising method when minimum distances are included in the objective. As minimum distances are much more sensitive to the exact composition of the core than mean distances, we need better methods in this case. The LR method seemed to be very well suited for these more difficult problems, and was often much better than the advanced REMC method. LR can become quite slow, especially in case of large datasets and intensities, but depending on the application this significant increase in minimum distance could be worth the extra runtime. However, in cases where high minimum distance is not required, the performance of LR over Local Search does not warrant the extra runtime required by LR.

### Mixed Replica Search motivation

Based on the results from the previous subsections we are now able to give further motivation for the specific composition of our new Mixed Replica search (MixRep) algorithm. We showed that the simple methods performed very well in many experiments, but it was not always the same method that was the most promising and each of the simple methods has its drawbacks. Local Search and MSTRAT clearly cannot cope with minimum distance measures, and both MSTRAT and LR Search become slower when run on relatively large datasets. Including several methods in one robust algorithm avoids the need of selecting the most suitable method. As Local Search is the fastest method and LR is better when including minimum distances in the objective, we decided to use both these methods in the initial search phase. However, the results showed that in some cases the advanced REMC slightly outperformed the other methods in terms of diversity scores. To benefit from advantages of both the simple methods and REMC we used a Mixed Replica approach, which contains LR and Local Search replicas at the start. Additional advanced search engines are then included in later stages of the search (MC & Tabu) to find better scores not obtainable by the simpler methods, if such scores are possible in the dataset. In this way our method will be able to tackle different problems in an efficient way, with fast computation on simple problems and yet very good results in more difficult settings, if additional runtime is available.

### Performance of Mixed Replica Search

Now we will present results for our new robust Mixed Replica search and compare these with the results of the original REMC Core Hunter algorithm. Table 5 shows diversity scores and runtimes for both methods with a sampling intensity of 20%, using the original Core Hunter evaluation measures, either single or mixed where the mixed measure again also includes both auxiliary measures (PN & CV). As we can see, MixRep always samples cores with equal or even slightly higher diversity scores than REMC within the applied time limit. The largest increase in diversity (about 2.5%) is observed when optimizing the mixed objective on the large pea dataset. More importantly, overall runtimes are significantly lower for the MixRep algorithm than for REMC.

**Table 5:** Results of Mixed Replica search vs. REMC – original measures (int = 0.2)

**Algorithm**^**∗**^	**MR**	**(t)**	**CE**	**(t)**	**SH**	**(t)**	**HE**	**(t)**	**NE**	**(t)**	**Mixed**^**∗∗**^	**(t)**
	**Bulk maize data set (275)**											
REMC	0.572	1.0s	0.641	2.0s	4.531	2.0s	0.667	1.0s	3.446	3.0s	10.680	15.0s
MixRep	0.572	0.45s	0.641	0.46s	4.531	0.49s	0.667	0.50s	3.446	0.59s	10.680	2.2s
Original	0.440		0.521		4.399		0.620		2.937			
	**Accession maize data set (521)**											
REMC	0.694	4.0s	0.752	4.0s	4.670	5.0s	0.676	3.0s	3.502	20.0s	11.086	50.1s
MixRep	**0.695**	1.1s	0.752	0.68s	4.670	1.1s	0.676	0.67s	3.502	3.8s	**11.087**	17.1s
Original	0.630		0.696		4.467		0.591		2.742			
	**Flax data set (708)**											
REMC	0.511	5.0s	0.511	4.0s	5.340	30.0s	0.262	4.0s	1.469	30.0s	8.874	60.4s
MixRep	**0.512**	1.6s	**0.512**	1.7s	5.340	0.80s	**0.263**	0.83s	1.469	1.6s	**8.878**	13.0s
Original	0.468		0.468		5.285		0.222		1.377			
	**Pea data set (1283)**											
REMC	0.591	50.0s	0.595	30.0s	3.553	7.0s	0.437	15.0s	1.865	15.0s	7.876	61.2s
MixRep	**0.593**	3.2s	**0.597**	3.1s	**3.556**	1.5s	**0.440**	1.4s	**1.867**	7.7s	**7.945**	37.6s
Original	0.509		0.515		3.482		0.381		1.713			
	**Large pea data set^▾^ (4429)**											
REMC	0.577	03m41s	0.580	08m49s	3.470	08m37s	0.448	04m29s	1.875	05m22s	7.621	10m03s
MixRep	**0.594**	01m18s	**0.596**	53.5s	**3.486**	39.6s	**0.465**	36.3s	**1.886**	47.3s	**7.811**	10m21s
Original	0.464		0.466		3.348		0.352		1.609			

^*^For each combination of algorithm, dataset and evaluation measure, 20 independent runs were performed from which averaged results are reported. By default runs were limited by a runtime of 60 seconds, except for the large pea dataset where a runtime limit of 10 minutes was applied.

^**^Results shown are those of a pseudo-index containing all seven measures with equal weights.

^▾^These results were computed on the helios server.

Results for the same experiments, but now with a sampling intensity of only 5%, are reported in Table 6 and for this lower sampling intensity the same observations hold. Here, in most cases MixRep and REMC sample cores with exactly the same diversity scores. Only for the large pea dataset is there a small but consistent improvement of MixRep over REMC. Again the most important observation is that MixRep is clearly the faster method. We conclude that for the original Core Hunter measures MixRep is much faster than REMC and yet samples cores with equal or sometimes slightly higher diversity scores. For these measures MixRep leads to results very similar to those of Local Search, which were discussed in the previous subsections, and therefore is generally faster than the other methods with only a small time overhead caused by the other replicas (besides Local Search) in the advanced MixRep algorithm.

**Table 6:** Results of Mixed Replica search vs. REMC – original measures (int = 0.05)

**Algorithm**^**∗**^	**MR**	**(t)**	**CE**	**(t)**	**SH**	**(t)**	**HE**	**(t)**	**NE**	**(t)**	**Mixed**^**∗∗**^	**(t)**
	**Bulk maize data set (275)**											
REMC	0.643	0.35s	0.700	0.35s	4.568	0.65s	0.685	0.55s	3.631	3.0s	10.790	7.0s
MixRep	0.643	0.19s	0.700	0.29s	4.568	0.50s	0.685	0.42s	3.631	2.0s	10.790	2.0s
Original	0.440		0.521		4.399		0.620		2.937			
	**Accession maize data set (521)**											
REMC	0.723	0.75s	0.782	2.0s	4.724	2.0s	0.702	5.0s	3.886	6.0s	11.216	50.0s
MixRep	0.723	0.38s	0.782	0.68s	4.724	0.83s	0.702	1.7s	**3.887**	3.5s	11.216	10.2s
Original	0.630		0.696		4.467		0.591		2.742			
	**Flax data set (708)**											
REMC	0.533	3.0s	0.533	3.0s	5.358	2.0s	0.278	3.0s	1.504	4.0s	8.965	30.0s
MixRep	0.533	0.81s	0.533	0.82s	5.358	0.76s	0.278	0.97s	1.504	1.5s	8.965	11.2s
Original	0.468		0.468		5.285		0.222		1.377			
	**Pea data set (1283)**											
REMC	0.626	10.0s	0.629	7.0s	3.578	2.0s	0.451	3.0s	1.898	2.0s	8.083	40.0s
MixRep	0.626	1.4s	0.629	1.2s	3.578	0.62s	0.451	0.55s	1.898	0.95s	**8.084**	11.0s
Original	0.509		0.515		3.482		0.381		1.713			
	**Large pea data set^▾^ (4429)**											
REMC	0.633	06m05s	0.634	36.8s	3.515	27.6s	0.492	13.9s	1.982	06m53s	8.147	09m36s
MixRep	**0.635**	7.5s	**0.637**	13.0s	**3.518**	2.1s	**0.496**	8.9s	**1.983**	9.6s	**8.158**	02m15s
Original	0.464		0.466		3.348		0.352		1.609			

^*^For each combination of algorithm, dataset and evaluation measure, 20 independent runs were performed from which averaged results are reported. By default runs were limited by a runtime of 60 seconds, except for the large pea dataset where a runtime limit of 10 minutes was applied.

^**^Results shown are those of a pseudo-index containing all seven measures with equal weights.

^▾^These results were computed on the helios server.

Our previous results showed that including the new minimum distance measures in the objective function, together with the original mean distances, can lead to cores with higher minimum distance while retaining similar means. Table 7 compares results of MixRep and REMC when optimizing such a mixed objective, which includes both mean and minimum MR with equal weight. With a sampling intensity of 20%, MixRep selects cores with higher minimum distances than REMC for all datasets, except the smallest maize (bulk) dataset for which scores are almost equal, while retaining similar mean distances. For the large pea dataset MixRep even leads to a larger mean component than REMC in addition to a significant increase in minimum score. The highest increase in mixed score (> 45%) is observed for the large pea dataset. This dataset, which is the largest of those tested, also has a relatively low dimension and thus presents a difficult problem when high minimum distances are desired. Runtimes of both methods are often similar. MixRep is occasionally faster than REMC, e.g. for the accession maize and flax datasets, but MixRep is slower for large datasets because of its LR component. For the large pea dataset, MixRep is significantly slower than REMC, but again here it does lead to the highest improvement.

**Table 7:** Results of Mixed Replica search vs. REMC – Mixed MR

**Optimized →**	MixedMR^**^(int=0.2)						MixedMR^**^(int=0.05)			
Algorithm^*^	**MixedMR**	**(t)**	**MRmin**^**∘**^	**MR**^**∘**^			**MixedMR**	**(t)**	**MRmin**^**∘**^	**MR**^**∘**^
	**Bulk maize data set (275)**									
REMC	**0.476**	23.6s	0.395	0.557			0.582	4.7s	0.534	0.629
MixRep	0.475	23.9s	0.393	0.557			0.582	5.8s	0.534	0.630
Original			0.116	0.440					0.116	0.440
	**Accession maize data set (521)**									
REMC	0.608	51.0s	0.526	0.690			**0.678**	56.0s	0.638	0.717
MixRep	**0.618**	6.9s	0.555	0.682			0.676	31.5s	0.635	0.717
Original			0.294	0.630					0.294	0.630
	**Flax data set (708)**									
REMC	0.422	39.5s	0.337	0.508			**0.487**	20.3s	0.446	0.529
MixRep	**0.440**	19.0s	0.378	0.502			0.486	44.5s	0.445	0.527
Original			0.000	0.468					0.000	0.468
	**Pea data set (1283)**									
REMC	0.396	02m27s	0.209	0.583			0.510	26.1s	0.397	0.622
MixRep	**0.454**	02m01s	0.324	0.583			**0.520**	6.6s	0.429	0.612
Original			0.000	0.509					0.000	0.509
	**Large pea data set^▾^ (4429)**									
REMC	0.278	40m29s	0.000	0.556			0.313	02m03s	0.000	0.626
MixRep	**0.405**	01h40m	0.230	0.580			**0.487**	02m48s	0.361	0.612
Original			0.000	0.464					0.000	0.464

^*^For each combination of algorithm, dataset and evaluation measure, 20 independent runs were performed from which averaged results are reported. By default runs were limited by a runtime of 60 seconds, with some exceptions. For the small pea dataset with an intensity of 20%, a runtime limit of 150 seconds was applied. For the large pea dataset runtime limits were set to 10 minutes for the 5% intensity and 2 hours for the 20% intensity.

^**^Results shown are those of a pseudo-index containing both minimum and mean MR distance, with equal weight = 0.5.

^∘^Components of mixed MR measure.

^▾^These results were computed on the helios server.

In case of a lower sampling intensity of only 5%, results of both methods are similar in most cases. Only for the pea datasets does MixRep lead to higher scores than REMC with a significant relative improvement in mixed score (> 55%) for the large pea set, caused by a large increase of the minimum component while again retaining most of the mean component. It is interesting to note that for this large pea dataset, REMC samples cores with zero minimum distance in all experiments, including some identical accessions in the core, while MixRep always leads to non-zero minimum distances. Similar results for a mixed measure containing both minimum and mean CE are reported in (see Additional file 1: Table S3).

We conclude that when aiming at high minimum distances the results of MixRep are very similar to those of the LR method and therefore often significantly better than those of all other methods (not only REMC, but also MSTRAT and Local Search as shown before). The new MixRep algorithm is often able to sample cores with much higher minimum distances than REMC, especially for these datasets where it is more difficult to reach these high minimum values e.g. datasets with large size and/or low dimension. However for these larger datasets, MixRep is slower than REMC but then gains in minimum distance are very high.

## Conclusions

Our results show that when aiming at core subsets in which all accessions are sufficiently distant from each other including minimum distance measures in the objective function, in combination with the original mean distance measures, improves performance. This additional measure often leads to cores with significantly higher minimum distances while retaining very similar mean distance scores compared to optimizing mean distance alone.

With Core Hunter II we have introduced a new advanced search algorithm – Mixed Replica search (MixRep) which uses heterogeneous replicas, an approach inspired by the results of a comparison of several algorithms – and showed that this new method improves on the results of the original REMC algorithm in two different ways. When optimizing the original Core Hunter evaluation measures (MR, CE, SH, HE, NE or a mixed measure) the new MixRep algorithm samples cores with equal or slightly higher diversity scores than REMC, while being much faster. Secondly, when minimum distances (MRmin or CEmin) are included in the objective to avoid selection of identical or very similar accessions inside the core, using MixRep instead of the original REMC often leads to significantly higher minimum distance scores. This effect is most obvious in case of large collections with relatively low dimension when sampling with fairly large intensities. For these large datasets, it does take more time to reach high minimum distances so it is important to apply higher runtime limits to achieve this goal. However the beauty of the MixRep algorithm is that in the case where minimum distances are not important, one can simply apply lower runtime limits and the same algorithm will quickly sample very good cores in terms of the remaining evaluation measures.

Future work concerning new versions of Core Hunter includes adding support for phenotypic variables, as for now only genetic marker data are supported. Furthermore, Core Hunter is currently freely available, but only as a command-line tool so development on a rich graphical user interface has already begun to provide user-friendly access to this core selection tool. Finally it might also be interesting to try to further improve results by plugging in new search engines inside the MixRep replicas. For example the current LR replica is quite slow for large core sizes, although including this replica does lead to significantly better results in terms of minimum distance scores. It may be useful to look for faster search replicas which also have this interesting property, to speed up the MixRep algorithm when aiming at high minimum distances.

## Endnotes

^a^Optimal only in theory for large datasets, where evaluating all possible subsets is computationally infeasible. In practice we turn to heuristic methods that cannot guarantee an optimal solution, to keep the search process feasible.^b^Because our distance measures cannot be computed on sets containing less than two accessions, we have slightly modified this approach by exhaustively selecting the best first pair and then proceeding with the LR scheme. Selecting two accessions by exhaustive search is still computationally feasible.^c^These same stop criteria are available for all randomized heuristics introduced in this paper.^d^Temperatures of newly created MC replicas are chosen randomly between a given minimum and maximum temperature. By default these are set to 50.0 and 100.0 respectively, and if desired the user can specify other minimum and maximum values using the advanced search options.^e^Modifications of this kind are frequently applied when using tabu search in practice, to avoid excessive memory usage and computation time.^f^As noted before, distance measures treat each allele as one dimension so the dimension of a dataset is defined as the total number of alleles over all marker loci.^g^The actual runtimes might slightly exceed this limit as the elapsed runtime is only checked after each search round and some algorithms implement quite intensive search rounds performing several search steps, possibly for several replicas.^h^Note that these convergence times are bounded by the runtime limit and it is always possible that further improved would have been made beyond this limit.^i^By identical we mean that the accessions can not be distinguished from one another using the available data. The accessions have the same alleles for all available markers used within the dataset.

## Competing interests

The authors declare that they have no competing interests.

## Authors’ contributions

HDB proposed the algorithm, implemented it, and performed all new experiments under the supervision of VF. PS provided data, advice on experiments and biological interpretation of the results. GD provided advice on algorithm development. HDB wrote the initial manuscript with all authors contributing to the final version. All authors read and approved the final manuscript.

## Supplementary Material

Additional file 1 “supplementary_results.pdf” — Supplementary results. This file contains some supplementary tables which are numbered with prefix S (S1, S2, etc.). These tables contain results of additional experiments that are similar to those which have been included in the main article itself (minimum versus mean CE). Click here for file

## References

[B1] Frankel OH Arber W, Illmensee K, Peacock WJ, Starlinger P Genetic perspectives of germplasm conservation Genetic manipulation: impact on man and society 1984 Cambridge: Cambridge University Press 161 170

[B2] Wright S Evolution and the Genetics of Populations: A treatise in four volumes, Volume IV 1978 1427 East 60th Street Chicago, IL 60637 USA: University of Chicago Press

[B3] Cavalli-Sforza L Edwards A Phylogenetic analysis. Models and estimation procedures Am J Human Genet 1967 19 233 257 6026583 PMC1706274

[B4] Shannon CE A mathematical theory of communication Bell Syst Tech J 1948 27 379 423

[B5] Berg EE Hamrick JL Quantification of genetic diversity at allozyme loci Can J Forest Res 1997 27 415 424 10.1139/x96-195

[B6] Franco J Crossa J Warburton ML Taba S Sampling strategies for conserving Maize diversity when forming core subsets using genetic markers Crop Sci 2006 46 854 864 10.2135/cropsci2005.07-0201

[B7] Kim KW Chung HK Cho GT Ma KH Chandrabalan D Gwag JG Kim TS Cho EG Park YJ PowerCore: a program applying the advanced M strategy with a heuristic search for establishing core sets Bioinformatics 2007 23 2155 2162 10.1093/bioinformatics/btm313 17586551

[B8] Wang JC Hu J Liu NN Xu HM Zhang S Investigation of combining plant genotypic values and molecular marker information for constructing core subsets J Integr Plant Biol 2006 48 11 1371 1378 10.1111/j.1744-7909.2006.00348.x

[B9] Franco J Crossa J Desphande S Hierarchical multiple-factor analysis for classifying genotypes based on phenotypic and genetic data Crop Science 2010 50 105 117 10.2135/cropsci2009.01.0053

[B10] Sokal R Michener C of Kansas U A statistical method for evaluating systematic relationships 1958 2502 Westbrooke Circle Lawrence KS 66045–4444: University of Kansas science bulletin, University of Kansas

[B11] Franco J Crossa J Taba S Shands H A multivariate method for classifying cultivars and studying group x environment x trait interaction Crop Sci 2003 43 1249 1258 10.2135/cropsci2003.1249

[B12] Franco J Crossa J The modified location model for classifying genetic resources. I. Association between categorial and continuous variables Crop Sci 2002 42 1719 1726 10.2135/cropsci2002.1719

[B13] Franco J Crossa J Taba S Eberhart SA The modified location model for classifying genetic resources. II Unrestrictive variance-covariance matrices Crop Sci 2002 42 1727 1736 10.2135/cropsci2002.1727

[B14] Franco J Crossa J Villaseñor J Taba S Eberhart SA A two-stage, three-way method for classifying genetic resources in multiple environments Crop Sci 1999 39 259 267 10.2135/cropsci1999.0011183X003900010040x

[B15] Franco J Crossa J Villaseñor J Taba S Eberhart SA Classifying genetic resources by categorical and continuous variables Crop Sci 1998 38 6 1688 1696 10.2135/cropsci1998.0011183X003800060045x

[B16] Brown AHD Core collections: a practical approach to genetic resources management Genome 1989 31 818 824 10.1139/g89-144

[B17] Franco J Crossa J Taba S Shands H A sampling strategy for conserving genetic diversity when forming core subsets Crop Sci 2005 45 1035 1044 10.2135/cropsci2004.0292

[B18] Schoen DJ Brown AH Conservation of allelic richness in wild crop relatives is aided by assessment of genetic markers Proc Nat Acad Sci 1993 90 10623 10627 10.1073/pnas.90.22.10623 8248153 PMC47829

[B19] Gouesnard B Bataillon TM Decoux G Rozale C Schoen DJ David JL MSTRAT: An algorithm for building germ plasm core collections by maximizing allelic or phenotypic richness J Heredity 2001 92 93 94 10.1093/jhered/92.1.93 11336240

[B20] Jansen J van Hintum T Genetic distance sampling: a novel sampling method for obtaining core collections using genetic distances with an application to cultivated lettuce TAG Theor Appl Genet 2007 114 421 428 10.1007/s00122-006-0433-9 17180377

[B21] Wang J Hu J Xu H Zhang S A strategy on constructing core collections by least distance stepwise sampling TAG Theor Appl Genet 2007 115 1 8 10.1007/s00122-007-0533-1 17404701

[B22] Thachuk C Crossa J Franco J Dreisigacker S Warburton M Davenport G Core Hunter: an algorithm for sampling genetic resources based on multiple genetic measures BMC Bioinf 2009 10 243 10.1186/1471-2105-10-243 PMC2734557 19660135

[B23] Kirkpatrick S Gelatt CD Vecchi MP Optimization by simulated annealing Science 1983 220 671 680 10.1126/science.220.4598.671 17813860

[B24] Reif JC Melchinger AE Frisch M Genetical and mathematical properties of similarity and dissimilarity coefficients applied in plant breeding and seed bank management Crop Sci 2005 45 1 7 10.2135/cropsci2005.0001

[B25] Ferri FJ Pudil P Hatef M Kittler J Gelsema ES, Kanal LN Comparative study of techniques for large-scale feature selection Pattern Recognition in Practice, IV: Multiple Paradigms, Comparative Studies and Hybrid Systems 1994 Amsterdam 403 413

[B26] Mitchell M An Introduction to Genetic Algorithms (Complex Adaptive Systems). A Bradford Book, third printing edition 1998 55 Hayward Street Cambridge, MA 02142-1493 USA: The MIT Press

[B27] Dubreuil P Warburton M Chastanet M Hoisington D Charcosset A More on the introduction of temperate maize into Europe: large-scale bulk SSR genotyping and new historical elements Maydica 2006 51 281 291

[B28] Warburton M Crossa J Diaz L Gomez A Taba S Diversidad genética en criollos de mais medida por microsatélites Congreso Nacional de Biotecnología Agropecuaria y Forestal 2004 Chapingo, México

[B29] Smýkal P Bačová-Kerteszová N Kalendar R Corander J Schulman AH Pavelek M Genetic diversity of cultivated flax (Linum usitatissimum L.) germplasm assessed by retrotransposon-based markers Theor Appl Genet 2011 122 1385 1397 10.1007/s00122-011-1539-2 21293839

[B30] Smýkal P Hýbl M Corander J Jarkovský J Flavell A Griga M Genetic diversity and population structure of pea (Pisum sativum L.) varieties derived from combined retrotransposon, microsatellite and morphological marker analysis Theor Appl Genet 2008 117 413 424 10.1007/s00122-008-0785-4 18504543

[B31] Jing R Vershinin A Grzebyta J Shaw P Smýkal P Marshall D Ambrose MJ Ellis THN Flavell AJ The genetic diversity and evolution of field pea (Pisum) studied by high throughput retrotransposon based insertion polymorphism (RBIP) marker analysis BMC Evolutionary Biol 2010 10 44 10.1186/1471-2148-10-44 PMC2834689 20156342

[B32] Smýkal P Kenicer G Flavell AJ Corander J Kosterin O Redden RJ Ford R Coyne CJ Maxted N Ambrose MJ Ellis THN Phylogeny, phylogeography and genetic diversity of the Pisum genus Plant Genet Res 2011 9 4 18 10.1017/S147926211000033X

