# Supplementary material for: Core Hunter II: fast core subset selection based on multiple genetic diversity measures using Mixed Replica search
Source: BMC Bioinformatics. 2012 Nov 23;13:312. doi: 10.1186/1471-2105-13-312 (PMC3554476; doi:10.1186/1471-2105-13-312)
Supplement: Additional file 1 — “supplementary_results.pdf” — Supplementary results. This file contains some supplementary tables which are numbered with prefix S (S1, S2, etc.). These tables contain results of additional experiments that are similar to those which have been included in the main article itself (minimum versus mean CE). [file 1471-2105-13-312-S1.pdf]

Supplementary Material

for

Core Hunter II: Fast core subset selection based on multiple genetic diversity measures using Mixed Replica Search

Table S1 - Comparison of REMC with simpler methods – minimum vs. mean CE (int = 0.2)

Comparison of the REMC algorithm with three more simple methods (Local Search, MSTRAT and LR(2,1)) optimizing mean and minimum CE distance. The sampling intensity is set to 0.2, resulting in core sets which contain 20% of the accessions of the original collection.

Table S2 - Comparison of REMC with simpler methods – minimum vs. mean CE (int = 0.05)

Comparison of the REMC algorithm with three more simple methods (Local Search, MSTRAT and LR(2,1)) optimizing mean and minimum CE distance. The sampling intensity is set to 0.05, resulting in core sets which contain 5% of the accessions of the original collection.

Table S3 - Results of Mixed Replica search vs. REMC – Mixed CE

This table compares results for the new Mixed Replica method with those of the original REMC, optimizing a mixed objective which contains both mean and minimum CE distance with equal weight, for both sampling intensities of 20% and 5%.

Table S1: Comparison of REMC with simpler methods – minimum vs. mean CE (int = 0.2)

Optimized →	CE		CEmin		MixedCE**		
Algorithm*	CE (t)	CEmin•	CEmin (t)	CE•	MixedCE (t)	CEmin°	CE°
Bulk maize data set (275)							
Local S.	0.641 0.55 s	0.303	0.471 4.3 s	0.618	**0.550** 4.1 s	0.471	0.629
MSTRAT	0.641 0.32 s	0.303	0.464 1.7 s	0.613	0.545 1.2 s	0.459	**0.631**
LR(2,1)	0.641 0.64 s	0.303	0.470 1.3 s	0.625	0.547 1.6 s	0.470	0.625
REMC	0.641 2.0 s	0.303	**0.474** 36.4 s	0.620	**0.550** 13.2 s	**0.472**	0.629
Original	0.521	0.155	0.155	0.521		0.155	0.521
Accession maize data set (521)							
Local S.	0.752 1.0 s	0.328	0.496 0.60 s	0.698	0.659 12.1 s	0.568	0.749
MSTRAT	0.752 1.7 s	0.328	0.489 0.62 s	0.697	0.649 5.3 s	0.548	**0.750**
LR(2,1)	0.752 2.9 s	0.328	**0.613** 5.0 s	0.730	**0.673** 6.5 s	**0.613**	0.734
REMC	0.752 4.0 s	0.328	0.594 57.1 s	0.721	0.672 58.4 s	0.599	0.744
Original	0.696	0.328	0.328	0.696		0.328	0.696
Flax data set (708)							
Local S.	**0.512** 2.1 s	0.223	0.218 0.70 s	0.468	0.409 4.7 s	0.306	**0.512**
MSTRAT	**0.512** 5.1 s	0.222	0.226 1.3 s	0.469	0.397 13.0 s	0.283	**0.512**
LR(2,1)	**0.512** 7.4 s	0.223	**0.377** 11.3 s	0.494	**0.443** 15.8 s	**0.386**	0.499
REMC	0.511 4.0 s	0.223	0.316 37.4 s	0.475	0.422 45.8 s	0.337	0.507
Original	0.468	0.000	0.000	0.468		0.000	0.468
Pea data set (1283)							
Local S.	**0.597** 2.7 s	0.000	0.000 0.10 s	0.513	0.305 7.5 s	0.012	**0.597**
MSTRAT	**0.597** 28.5 s	0.000	0.000 0.74 s	0.514	0.304 60.7 s	0.012	0.596
LR(2,1)	**0.597** 34.3 s	0.000	**0.324** 59.0 s	0.576	**0.456** 01 m 20 s	**0.324**	0.587
REMC	0.595 30.0 s	0.000	0.006 22.0 s	0.514	0.387 58.1 s	0.187	0.587
Original	0.515	0.000	0.000	0.515		0.000	0.515
Large pea data set▼ (4429)							
LR(2,1)	**0.596** 42 m 35 s	0.000	**0.243** 53 m 40 s	0.558	**0.412** 01 h 39 m	**0.243**	**0.582**
REMC	0.580 08 m 49 s	0.000	0.000 0.19 s	0.467	0.275 09 m 11 s	0.000	0.550
Original	0.466	0.000	0.000	0.466		0.000	0.466

* For each combination of algorithm, dataset and evaluation measure, 20 independent runs were performed from which averaged results are reported. By default runs were limited by a runtime of 60 seconds, except for the large pea dataset where a runtime limit of 10 minutes was applied. Furthermore the LR method does not accept a runtime limit but continues search until the desired core size has been reached.

** Results shown are those of a pseudo-index containing both minimum and mean CE distance, with equal weight = 0.5.

• Not used during optimization, but computed afterwards on the constructed core sets.

° Components of mixed CE measure.

▼ These results were computed on the helios server.

Table S2: Comparison of REMC with simpler methods – minimum vs. mean CE (int = 0.05)

Optimized →	CE		CEmin		MixedCE**		
Algorithm*	CE (t)	CEmin•	CEmin (t)	CE•	MixedCE (t)	CEmin°	CE°
Bulk maize data set (275)							
Local S.	**0.700** 0.25 s	0.537	0.590 0.50 s	0.673	0.640 0.61 s	0.588	0.693
MSTRAT	0.699 0.14 s	0.514	0.579 0.44 s	0.666	0.635 0.36 s	0.572	**0.697**
LR(2,1)	**0.700** 0.37 s	0.565	0.591 0.57 s	0.677	0.633 0.77 s	0.573	0.694
REMC	**0.700** 0.35 s	0.565	**0.605** 2.8 s	0.691	**0.647** 11.6 s	**0.604**	0.690
Original	0.521	0.155	0.155	0.521		0.155	0.521
Accession maize data set (521)							
Local S.	0.781 0.35 s	0.566	0.603 0.21 s	0.713	0.726 1.6 s	0.676	0.776
MSTRAT	0.781 0.27 s	0.557	0.578 0.29 s	0.699	0.721 0.75 s	0.666	**0.777**
LR(2,1)	0.781 1.2 s	0.576	0.692 2.0 s	0.768	0.723 2.4 s	0.681	0.766
REMC	**0.782** 2.0 s	0.576	**0.700** 47.0 s	0.765	**0.735** 60.0 s	**0.697**	0.772
Original	0.696	0.328	0.328	0.696		0.328	0.696
Flax data set (708)							
Local S.	0.533 1.0 s	0.341	0.310 0.20 s	0.470	0.475 4.1 s	0.419	**0.532**
MSTRAT	0.533 0.67 s	0.343	0.311 0.39 s	0.469	0.469 1.6 s	0.406	**0.532**
LR(2,1)	0.533 3.3 s	0.357	**0.446** 4.3 s	0.515	0.481 6.3 s	**0.446**	0.517
REMC	0.533 3.0 s	0.337	0.429 39.2 s	0.504	**0.487** 19.1 s	**0.446**	0.529
Original	0.468	0.000	0.000	0.468		0.000	0.468
Pea data set (1283)							
Local S.	0.629 1.2 s	0.261	0.105 0.10 s	0.514	0.486 8.2 s	0.344	0.627
MSTRAT	0.629 1.6 s	0.255	0.100 0.29 s	0.517	0.456 4.6 s	0.284	**0.628**
LR(2,1)	0.629 3.5 s	0.261	**0.434** 6.0 s	0.600	**0.526** 8.0 s	**0.434**	0.617
REMC	0.629 7.0 s	0.261	0.351 48.3 s	0.563	0.512 57.0 s	0.398	0.625
Original	0.515	0.000	0.000	0.515		0.000	0.515
Large pea data set▼ (4429)							
LR(2,1)	**0.637** 01 m 19 s	0.000	**0.357** 01 m 55 s	0.600	**0.491** 03 m 08 s	**0.367**	0.615
REMC	0.634 36.8 s	0.000	0.000 0.13 s	0.468	0.314 03 m 47 s	0.000	**0.628**
Original	0.466	0.000	0.000	0.466		0.000	0.466

* For each combination of algorithm, dataset and evaluation measure, 20 independent runs were performed from which averaged results are reported. By default runs were limited by a runtime of 60 seconds, except for the large pea dataset where a runtime limit of 10 minutes was applied. Furthermore the LR method does not accept a runtime limit but continues search until the desired core size has been reached.

** Results shown are those of a pseudo-index containing both minimum and mean CE distance, with equal weight = 0.5.

• Not used during optimization, but computed afterwards on the constructed core sets.

° Components of mixed CE measure.

▼ These results were computed on the helios server.

Table S3: Results of Mixed Replica search vs. REMC – Mixed CE

Optimized →	MixedCE** (int=0.2)			MixedCE** (int=0.05)		
Algorithm*	MixedCE (t)	CEmin°	CE°	MixedCE (t)	CEmin°	CE°
			Bulk maize data set (275)			
REMC	0.550 13.2 s	0.472	0.629	0.647 11.6 s	0.604	0.690
MixRep	0.550 3.2 s	0.472	0.629	**0.648** 3.0 s	0.605	0.691
Original		0.155	0.521		0.155	0.521
			Accession maize data set (521)			
REMC	0.672 58.4 s	0.599	0.744	**0.735** 60.0 s	0.697	0.772
MixRep	**0.674** 7.7 s	0.613	0.736	0.734 28.8 s	0.694	0.773
Original		0.328	0.696		0.328	0.696
			Flax data set (708)			
REMC	0.422 45.8 s	0.337	0.507	**0.487** 19.1 s	0.446	0.529
MixRep	**0.441** 21.0 s	0.381	0.500	0.486 45.8 s	0.445	0.527
Original		0.000	0.468		0.000	0.468
			Pea data set (1283)			
REMC	0.404 02 m 25 s	0.221	0.588	0.512 57.0 s	0.398	0.625
MixRep	**0.455** 02 m 12 s	0.325	0.586	**0.525** 7.0 s	0.436	0.614
Original		0.000	0.515		0.000	0.515
			Large pea data set▼ (4429)			
REMC	0.279 33 m 46 s	0.000	0.559	0.314 03 m 47 s	0.000	0.628
MixRep	**0.406** 01 h 42 m	0.230	0.582	**0.490** 02 m 49 s	0.366	0.615
Original		0.000	0.466		0.000	0.466

* For each combination of algorithm, dataset and evaluation measure, 20 independent runs were performed from which averaged results are reported. By default runs were limited by a runtime of 60 seconds, with some exceptions. For the small pea dataset with an intensity of 20%, a runtime limit of 150 seconds was applied. For the large pea dataset runtime limits were set to 10 minutes for the 5% intensity and 2 hours for the 20% intensity.

** Results shown are those of a pseudo-index containing both minimum and mean CE distance, with equal weight = 0.5.

° Components of mixed CE measure.

▼ These results were computed on the helios server.
